# Regulation of Inflammation by Adenosine

**DOI:** 10.3389/fimmu.2013.00085

**Published:** 2013-04-08

**Authors:** György Haskó, Bruce Cronstein

**Affiliations:** ^1^Department of Surgery, New Jersey Medical School, University of Medicine and Dentistry of New JerseyNewark, NJ, USA; ^2^Department of Medicine, New York University School of MedicineNew York, NY, USA

**Keywords:** monocytes, macrophages, adenosine, adenosine receptors, neutrophils

## Abstract

Adenosine, a purine nucleoside generated by the dephosphorylation of adenine nucleotides, is a potent endogenous physiologic and pharmacologic regulator of many functions. Adenosine was first reported to inhibit the inflammatory actions of neutrophils nearly 30 years ago and since then the role of adenosine and its receptors as feedback regulators of inflammation has been well established. Here we review the effects of adenosine, acting at its receptors, on neutrophil and monocyte/macrophage function in inflammation. Moreover, we review the role of adenosine in mediating the anti-inflammatory effects of methotrexate, the anchor drug in the treatment of Rheumatoid Arthritis and other inflammatory disorders.

## Introduction

**Figure 1:**
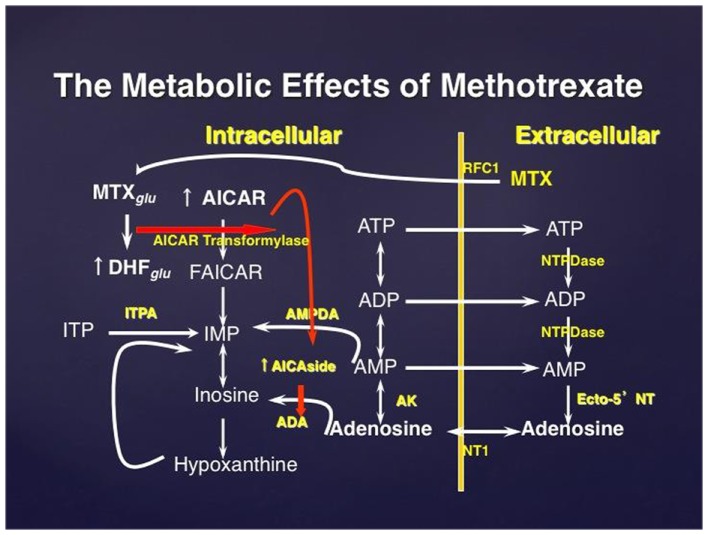
**The effect of methotrexate on adenosine release**. Methotrexate is actively transported into the cell where it is polyglutamated; MTX polyglutamate is a potent inhibitor of AMP deaminase. Accumulation of AICAR, an intermediate metabolite in de novo purine biosynthesis, leads to enhanced release of adenine nucleotides which are released into the extracellular space and converted to adenosine. MTXglu, methotrexate polyglutamates; DHFglu, dihydrofolate polyglutamates; AICAR, aminoimidazole carboxamidoribonucleotide; FAICAR, formyl AICAR; RFC1, reverse folate carrier 1; ADA, adenosine deaminase; AK, adenosine kinase; NTPDase, nucleoside triphosphate phosphohydrolase; ecto-5′NT, ecto-5′nucleotidase.

The nucleoside adenosine is a potent physiologic and pharmacologic regulator that is produced by cells in response to stress by breakdown of adenosine triphosphate (ATP) (Hasko and Cronstein, 2004; Sitkovsky et al., 2004; Hasko et al., 2005, 2008). ATP is broken down both intracellularly and extracellularly to generate adenosine (Figure 1). Intracellular adenosine is exported from cells via equilibrative nucleoside transporters or during apoptosis or necrosis. ATP and its degradation product adenosine diphosphate (ADP) are released from cells through a variety of mechanisms, including membrane damage, through connexin/pannexin and other channels, and through protein or hormone-transporting vesicles. When ATP and ADP are released, the phosphate groups of extracellular ATP and ADP are sequentially hydrolyzed, first by ecto-nucleoside triphosphate diphosphorylases (NTPDases, including CD39) and then by ecto-5′-nucleotidase (Ecto-5′NTase, CD73) (Yegutkin, 2008).

Extracellular levels of adenosine can rise from low nano-molar to micro-molar concentrations in response to stress (Hasko et al., 2008). Adenosine regulates cell function through ligation of adenosine receptors, which consist of a family of four cell surface 7-transmembrane receptors (A_1_R, A_2A_R, A_2B_R, and A3) (Linden, 2001). The activation of A_1_ and A_3_ receptors leads to decreased intracellular cyclic adenosine monophosphate (Gallardo-Soler et al., 2008) levels by coupling to pertussis toxin-inhibited G_i_-coupled signal transduction proteins. A_2A_ receptors are Gα_S_- or Gα_olf_-linked receptors that activate adenylyl cyclase, increase cAMP, and activate protein kinase A (PKA) and Epac1/2which activate their own signaling cascades to regulate cellular function. Interestingly, A_2A_ receptors can also signal in a G protein-independent manner. A_2B_ receptors can signal through both Gα_S_ and Gq proteins.

## Adenosine and the Signs of Inflammation

Classically, inflammation is characterized by rubor (redness), tumor (swelling), calor (heat), and functio laesa (loss of function). These manifestations of inflammation result principally from vascular dilatation and leakage and, although a large number and variety of mediators are involved in inflammation it is likely that adenosine, released at sites of tissue injury, plays a role in the pathogenesis or regulation of these signs. Adenosine, acting primarily at A_2A_ receptors, has long been known to be a potent vasodilator (Drury and Szent-Gyorgi, 1929) and this is the basis for use of adenosine and adenosine A_2A_ receptor agonists for pharmacologic stress testing. Thus, it is likely adenosine release at inflamed sites contributes to the erythema (rubor) and resulting heat loss (calor) associated with inflammation. Interestingly, diminished production of adenosine leads to dramatic vascular leakage resulting from diminished activation of adenosine A_2B_ receptors on the vascular endothelium (Thompson et al., 2004; Eckle et al., 2008) suggesting that the adenosine released at inflamed sites diminishes the swelling (tumor) that is so prominent at inflamed sites.

## Adenosine Inhibits Recruitment and Activation of Neutrophils

Neutrophils are recruited to inflamed sites by a combination of chemokines and adhesive interactions between leukocytes and the vascular endothelium. Adenosine diminishes inflammation by diminishing leukocyte recruitment; adenosine inhibits stimulated neutrophil adhesion to the vascular endothelium (Cronstein et al., 1986) and neutrophil-mediated injury to the endothelium. Adenosine receptor stimulation diminishes neutrophil adhesion to the endothelium by inhibiting both selectin- and integrin-mediated adhesive events (Cronstein et al., 1992; Bullough et al., 1995; Bouma et al., 1996; Sullivan et al., 2004). Presumably these same mechanisms apply to recruitment of other cell types to inflamed sites as well. Although having noted these anti-inflammatory effects of adenosine it has recently been reported that neutrophils release ATP which is converted to adenosine extracellularly and the adenosine binds to A_3_ receptors to promote chemotaxis and loss or inhibition of A_3_ receptors markedly reduces leukocyte recruitment to sites of bacterial infection (Chen et al., 2006). Other studies suggest that the A_3_ receptor-mediated effects on neutrophil recruitment are more selective and may not be important for recruitment to other chemoattractants (Montesinos et al., 2006).

Adenosine diminishes stimulated neutrophil production of oxygen radicals and other potentially deleterious mediators (Reviewed in Taylor et al., 2005). Moreover, adenosine, acting primarily at A_2A_ receptors inhibits phagocytosis of particles (Reviewed in Taylor et al., 2005). Although cAMP mediates many of the downstream effects of adenosine A_2A_ receptors via activation of PKA there have been reports that cAMP-PKA-independent mechanisms mediate inhibition of neutrophil activation by adenosine A_2A_ receptors.

## Adenosine and Classical Macrophage Activation

Macrophages are best known for initiating an effective innate immune response against microbes by recognizing pathogen-associated molecular patterns (PAMPs) through pattern-recognition receptors (PRRs) (Pozzi et al., 2005). Following phagocytosis, macrophages destroy most micro-organisms. By producing diverse molecules and presenting antigens to T cells, macrophages in addition to dendritic cells, orient the adaptive immune response leading to the expansion and differentiation of lymphocytes specific for invaders or cancer cells (Gordon and Taylor, 2005; Preynat-Seauve et al., 2006).

Macrophages comprise a heterogeneous population of cells, and show bewildering functional plasticity in response to dynamic micro-environmental cues. Macrophage heterogeneity arises as macrophages differentiate from immature monocyte precursors or yolk-sac macrophages (Mills et al., 2000; Kuroda et al., 2002; Mosser, 2003; Murray and Wynn, 2011). In a conscious parallel with T helper (Th)1 and Th2 lymphocytes, macrophages have been classified into M1 and M2 phenotypes. M1 or “classical” activation of macrophages is induced by toll-like receptor (TLR) agonists, either with or without the Th1 cytokine interferon (IFN)-γ, and results in an inflammatory phenotype characterized by expression of a series of inflammatory cytokines and chemokines, including tumor necrosis factor (TNF)-α, interleukin (IL)-1-β, IL-6, IL-12, and macrophage inflammatory protein (MIP)-1α (Mosser and Edwards, 2008; Biswas and Mantovani, 2010). M1 macrophages are strong promoters of Th1 immune responses (Hasko et al., 2000) and have anti-proliferative and cytotoxic activities, which result from their ability to produce reactive oxygen and nitrogen species, such as hydrogen peroxide, superoxide, nitric oxide (NO), and peroxynitrite, and pro-inflammatory cytokines.

The role of adenosine in regulating classical macrophage activation has been studied in detail. As such, adenosine has been shown to be a broad inhibitor of the pro-inflammatory consequences of classical macrophage activation. The anti-inflammatory effects of adenosine on M1 macrophages include suppression of cytokine/chemokine production (Hasko et al., 1996, 1998, 2007; Szabo et al., 1998; Xaus et al., 1999; Sipka et al., 2005, 2007; Ryzhov et al., 2008; Koscso et al., 2012) and NO production (Csoka et al., 2007a; Ryzhov et al., 2008). In contrast to the suppressive effect of adenosine on the production of pro-inflammatory mediators, adenosine augments production of the anti-inflammatory cytokine IL-10 by M1 macrophages. The current consensus is that the regulatory effects of adenosine on M1 macrophages are mediated predominantly by A_2A_ receptors (Hasko et al., 1996, 2009; Pinhal-Enfield et al., 2003; Nemeth et al., 2005; Kreckler et al., 2006; Csoka et al., 2007b; Chen et al., 2009; Wilson et al., 2009; Belikoff et al., 2011). For example, using A_2A_ receptor deficient mice combined with pharmacologic approaches, it has been shown that adenosine inhibits TNF-α, IL-6, and IL-12 release and augments IL-10 production by lipopolysaccharide (LPS)- or bacteria-activated macrophages mostly through A_2A_ receptors (Nemeth et al., 2005; Hasko et al., 2007; Kara et al., 2010a). Although, A_2B_ receptors have been overshadowed by A_2A_ receptors as the primary adenosine receptors shaping the function of M1 macrophages, there is growing evidence that A_2B_ receptors can also become operational in regulating M1 macrophage function. In this context, we recently showed that A_2B_ receptors but not A_2A_ receptors augment LPS-induced IL-10 production by RAW264.7 macrophages, which express high levels of A_2B_ receptors and low levels of A_2A_ receptors (Pinhal-Enfield et al., 2003). Moreover, adenosine can suppress LPS-induced TNF-α production even in A_2A_ receptor deficient mice, and this effect is mediated by A_2B_ receptors (Kara et al., 2010a). A_1_ adenosine receptors and A_3_ receptors are expressed at much lower levels on the surface of macrophages and their role in regulating macrophage function remains incompletely understood (Hasko et al., 1996).

Studies utilizing A_1_ receptor deficient mice recently showed that A_1_ receptors play a critical role in osteoclast development from monocytic precursors (Merrill et al., 1995). Interestingly, A_1_ receptors also regulate fusion of human peripheral blood monocytes into giant cells *in vitro* as well although the mechanism for this regulation has not been fully established (Merrill et al., 1997; Hasko and Pacher, 2012). Genetic studies have yet to confirm the role of A_3_ receptors in governing macrophage function (Nelms et al., 1999).

## Adenosine and Alternative Macrophage Activation

M2 or “alternatively activated” macrophages were originally described as macrophages induced by the Th2 cytokines IL-4 and IL-13. The effects of IL-4 and IL-13 on macrophages partially overlap because they use dimeric receptors that share the IL-4 receptor (IL-4R)α subunit. In contrast, the differences between signaling by IL-4 and IL-13 stem from the fact that while IL-4 is able to activate both the IL-4Rα/common γ chain and IL-4Rα/IL-13Rα dimers, IL-13 can only activate the latter complex. The intracellular signaling pathways are incompletely characterized and involve members of the Janus-activated kinase (JAK) and signal transducer and activator of transcription (STAT) family, especially STAT6. In addition to STAT6 (Gray et al., 2005), recent studies have identified CCAAT-enhancer-binding protein (C/EBP)β (Pauleau et al., 2004; Albina et al., 2005; Ruffell et al., 2009), cAMP response element-binding protein (CREB) (Odegaard et al., 2007), peroxisome proliferator-activated receptor (PPAR)γ (Gallardo-Soler et al., 2008; Satoh et al., 2010; Szanto et al., 2010), IFN regulatory factor (IRF)4 (El Chartouni et al., 2010; Liao et al., 2011), Krüppel-like factor 4 (KLF4) (Takeda et al., 2010), hypoxia-inducible factor-2 (HIF-2) (Pello et al., 2012), and c-MYC (Sica and Mantovani, 2012) as contributors to the transcriptional response driving M2 macrophage activation. Hallmark M2 markers include arginase-1, tissue inhibitor of metalloproteinases (TIMP)-1, macrophage galactose-type C-type lectin (mgl)-1, IL-4Rα, Ym1, and resistin-like molecule (RELM)α (Anthony et al., 2006). Increasingly, activation of multiple markers is used to unequivocally identify M2 macrophages in the context of responses to different antigens (Chen et al., 2012).

M2 macrophages are elicited following infection with multicellular parasites, and can lead to an inflammatory response qualitatively different from and capable of downregulating harmful Th1-type inflammatory responses. Recent studies have suggested that increased M2 macrophage arginase activity during helminth infections is an important element in the control and expulsion of worms (Hesse et al., 2001; Gordon, 2003; Edwards et al., 2006). M2 macrophages contribute to the control of inflammation and can mediate enhanced wound healing through arg-1-mediated production of collagen and insulin-like growth factor 1 (IGF-1), and by contributing to the clearance of cellular debris through scavenger receptors (Gordon, 2003; Anthony et al., 2006). The immunoregulatory/protective, rather than tissue damaging, role of M2 macrophages is also exemplified by the fact that they are abundant in healthy tissues that are associated with naturally immune suppressed states, such as placenta, lung, and other immunologically privileged sites (Noel et al., 2004). In contrast to their protective effects in acute inflammation, it has been proposed that M2 macrophages activated by IL-4 and IL-13 during asthma and chronic obstructive pulmonary disease contribute significantly to airway remodeling and lung fibrosis leading to lung dysfunction (Mantovani et al., 2004; Van Ginderachter et al., 2006; Martinez et al., 2008; Csoka et al., 2012). Additionally, M2 macrophages have also been shown to be hijacked by tumor cells to function as suppressors of anti-tumor T cell responses and stimulators of tumor angiogenesis (Martinez et al., 2008; Koroskenyi et al., 2011). Based on these observations, M2 macrophages have been proposed as an emerging therapeutic target for a variety of disease states.

We have recently discovered that adenosine strongly promotes IL-4/IL-13-induced M2 macrophage activation *in vitro*, as indicated by upregulation of the arginase-1, TIMP-1, and mgl-1 (Barczyk et al., 2010). Our studies, utilizing both pharmacological approaches and macrophages from adenosine receptor deficient and wild type (WT) mice, indicate that A_2B_ adenosine receptors, and to a lesser degree other adenosine receptors, are required for mediating the stimulatory effect of adenosine on IL-4-induced M2 macrophage activation. Our data also indicate that the stimulatory effect of adenosine receptor activation on M2 macrophage development is mediated by the transcription factor C/EBPβ, but not STAT6 or CREB (Barczyk et al., 2010).

While the designation M2 usually denotes macrophages activated by IL-4 or IL-13, M2 macrophages can also be induced by other anti-inflammatory stimuli, which include immune complexes, and heterogeneous deactivating mediators such as apoptotic cells, glucocorticoids, and IL-10 (Kular et al., 2012). Thus, IL-4/IL-13-activated macrophages are also called M2a, immune complex-activated macrophages are referred to as M2b, and macrophages activated by apoptotic cells, glucocorticoids, and IL-10 are termed M2c. In macrophages phagocytosing apoptotic cells, adenosine released endogenously activates A_2A_ receptors and inhibits the generation of the pro-inflammatory chemokines MIP-2 and cytokine-induced neutrophil-attracting chemokine (KC) (Kara et al., 2010b). Moreover, glucocorticoids promote survival of anti-inflammatory monocytes via upregulation and autocrine activation of A_3_ adenosine receptors (Mediero et al., 2012a). Together, in macrophages exposed to apoptotic cells and glucocorticoids adenosine switches macrophage phenotype from pro-inflammatory to anti-inflammatory (Nelms et al., 1999).

## Adenosine and Myeloid/Monocyte-Derived Syncytial Cells (Osteoclasts and Giant Cells)

Myeloid precursors can, in response to M-CSF and RANKL, differentiate into osteoclasts, multinucleated giant cells that mediate bone resorption (Mediero et al., 2012b). Studies utilizing A_1_ receptor deficient mice recently showed that A_1_ receptors play a critical role in osteoclast development from monocytic precursors and bone resorption (Ernst et al., 2010; McNally and Anderson, 2011). In contrast to A_1_ adenosine receptors, A_2A_ receptors inhibit osteoclast differentiation and function (Deaglio et al., 2007) and A_2A_ receptor stimulation has been shown to inhibit wear particle-induced osteolysis, a form of inflammatory bone destruction resulting from particulates shed from joint prostheses (Chalmin et al., 2012).

Similar to osteoclasts, in response to IFN-γ or other stimuli, monocytes will fuse to form multinucleated giant cells, hallmarks of responses to foreign bodies and such diseases as Sarcoidosis (Semenza, 2010). Interestingly, A_1_ receptors also regulate fusion of human peripheral blood monocytes into giant cells *in vitro* as well although the mechanism for this regulation has not been fully established (Merrill et al., 1997; Hasko and Pacher, 2012). Genetic studies have yet to confirm the role of A_3_ receptors in governing macrophage function (Nelms et al., 1999).

## Adenosine and T Cells

Forkhead box P3 (FOXP3)-expressing regulatory T (Treg) cells are crucial in the maintenance of immunological self-tolerance and in the regulation of immune responses. CD39 and CD73 are expressed on the surface of Foxp3^+^ Tregs and are increasingly used as markers of Tregs (Leibovich et al., 2002). Deaglio et al. (2007) and Adair (2005) showed that CD39 and CD73 on the surface of Tregs produce adenosine, which mediates a substantial portion of the anti-inflammatory and immune regulatory effects of Tregs by engaging A_2A_ receptors on effector T cells. More recently, Th 17 cells were also shown to express CD39 and CD73, which, by producing adenosine, suppress both CD4^+^ and CD8^+^ T cell effector functions (Hasko et al., 2009). The expression of both CD39 and CD73 on Th17 cells was upregulated by IL-6 and TGF-β, factors that are crucial for Th17 cell development (Ramanathan et al., 2009).

## Adenosine and the Angiogenic Switch in Macrophages

Vascular endothelial growth factor (VEGF) is a potent stimulator of angiogenesis and is crucial for the differentiation of endothelial cells during vasculogenesis, and for the outgrowth of new capillaries from pre-existing blood vessels (Ramanathan et al., 2007). VEGF is thus an important component of tissue repair, and is critical for the resolution of inflammation and wound healing. Macrophages are prominent producers of VEGF during the resolution of inflammation and wound healing. There is a plethora of evidence demonstrating that adenosine promotes angiogenesis, in a large part, by increasing macrophage VEGF production (Murphree et al., 2005; Csoka et al., 2007b; Ernens et al., 2010). It has been shown that adenosine stimulation of A_2A_ receptors on TLR-activated macrophages results in a switch from the production of inflammatory cytokines such as TNF-α and IL-12, to the production of anti-inflammatory and angiogenic factors, including IL-10 and VEGF (Ohta and Sitkovsky, 2001; Gessi et al., 2010) and we termed this process angiogenic switch (Csoka et al., 2007b). This model provides a sequential pathway whereby macrophages initially mediate inflammation through TLR-dependent activation to an M1 phenotype, but are then switched into an angiogenic phenotype by adenosine generated in response to hypoxia/ischemia within the wound area. In addition, the initial activation of macrophages by TLR agonists, which markedly induce expression of adenosine A_2A_ and A_2B_ adenosine receptors, primes these macrophages to respond to increased local levels of extracellular adenosine (Chan and Cronstein, 2010; Gessi et al., 2010). It is noteworthy that while A_2A_ (Csoka et al., 2007b) and A_2B_ (unpublished data) receptors mediate the angiogenic switch in murine macrophages, the adenosine-mediated increase in VEGF secretion by human monocytes is mediated by A_2A_, A_2B_, and A_3_ receptors (Varani et al., 2009, 2011).

## Adenosine and Adenosine Receptors in Inflammatory Diseases

From its initial identification as an anti-inflammatory ligand adenosine was thought to be an important endogenous feedback regulator of inflammation and tissue injury. Nonetheless, the first actual demonstration that adenosine, acting at A_2A_ receptors, was an endogenous anti-inflammatory agent had to wait until the development of adenosine receptor knockout mice. Liver injury in response to concanavalin A, a model for viral hepatitis, was markedly enhanced in the absence of adenosine A_2A_ receptors (Khoa et al., 2006), consistent with the hypothesis that, at least in the liver, increased adenosine release at inflamed sites suppresses inflammation and inflammatory injury.

However, making use of adenosine as an anti-inflammatory agent has remained more of a challenge due to the myriad other effects of adenosine acting at A_2A_ and other adenosine receptors, e.g., hypotension. Interestingly, it is now clear that low-dose methotrexate, the anchor drug for the treatment of rheumatoid arthritis, mediates its anti-inflammatory effects via promotion of adenosine release at inflamed sites (Reviewed in Khoa et al., 2001). The “immunosuppressive” effects of methotrexate-mediated inhibition of T cell proliferation are unlikely to account for the effects of methotrexate administered at doses well below those required to inhibit cellular proliferation (15–20 mg/week) and methotrexate is usually accompanied by folic acid or folinic acid supplementation to prevent toxicity without diminishing efficacy. The effects of adenosine, described above, on lymphocyte function provide a better explanation for the immunosuppressive effects of methotrexate, as used to treat Rheumatoid Arthritis (Khoa et al., 2001). Because adenosine receptor expression is increased on leukocytes in patients with Rheumatoid Arthritis (Levy et al., 2006; Hesdorffer et al., 2012), most likely the result of exposure of these cells to high concentrations of TNFα, a cytokine previously demonstrated to increase adenosine A_2A_ receptor expression and function (Smail et al., 1992; Thammavongsa et al., 2009), it is likely that these patients are “primed” to respond to increased adenosine concentrations resulting from methotrexate therapy.

## Adenosine and Aging

Immunologic and inflammatory responses are blunted at the beginning and end of life leading to increased susceptibilities to infection for neonates and the elderly. Recent work has suggested that adenosine and its receptors play a role in suppressing responses to infection. Levy et al. (2006) have reported that monocyte/macrophages from neonates are much more sensitive to adenosine A_3_ receptor-mediated suppression of inflammatory responses (TNF production) than those from adults. In contrast, lymphocytes from the elderly release increased amounts of adenosine leading to suppression of T cell responses (Hesdorffer et al., 2012).

## Adenosine is a Virulence Factor Produced by Pathogens

It is not uncommon for invasive pathogens to take advantage of mammalian mechanisms for suppression of host responses to promote spread or survival of the infecting organism. Thus, it was not surprising to find that *Candida albicans* hyphae release adenosine which suppresses neutrophil-mediated killing of the organism (Smail et al., 1992). More recent studies have demonstrated that *Staphylococcus aureus* also produce adenosine to avoid killing by the host as well (Thammavongsa et al., 2009). Thus, adenosine, produced by invasive organisms can promote spread of the organism by suppressing host killing of the bacteria.

## Conclusion

Adenosine is a potent endogenous anti-inflammatory agent that regulates the function of inflammatory cells via interaction with specific receptors expressed on these cells (Table 1). Already known as an endogenous regulator of inflammation, adenosine also mediates the anti-inflammatory effects of methotrexate, one of the most widely used anti-inflammatory drugs.

**Table 1:** **Cellular expression of adenosine receptors**.

	A_1_ receptor	A_2A_ receptor	A_2B_ receptor	A_3_ receptor
Neutrophils		Diminished adhesion, activation		Stimulates chemotaxis
M1 macrophages		Inhibit phagocytosis, inflammatory cytokine production, increase IL-10	Stimulates IL-10	
M2 macrophages		Stimulates M2 macrophage differentiation Production of pro-angiogenic factors		
Lymphocytes		Inhibits TH17 differentiation Stimulates T_Reg_ differentiation		
Osteoclasts and giant cells	Promotes osteoclast differentiation Stimulates giant cell formation	Inhibits osteoclast differentiation		

## Conflict of Interest Statement

Bruce Cronstein, Intellectual Property: Patents on use of adenosine A2A receptor agonists to promote wound healing and use of A2A receptor antagonists to inhibit fibrosis. Patent on use of adenosine A1 receptor antagonists to treat osteoporosis and other diseases of bone. Patent on use of adenosine A2A agonists to promote bone regeneration. Patent on use of anti-netrin-1 antibodies for the treatment of bone disease. Patent on use of adenosine A2A agonists and A1 antagonists to inhibit wear part.
